# Structure of liposome encapsulating proteins characterized by X-ray scattering and shell-modeling

**DOI:** 10.1107/S0909049513020827

**Published:** 2013-09-26

**Authors:** Mitsuhiro Hirai, Ryota Kimura, Kazuki Takeuchi, Yoshihiko Hagiwara, Rika Kawai-Hirai, Noboru Ohta, Noriyuki Igarashi, Nobutaka Shimuzu

**Affiliations:** a Graduate School of Engineering, Gunma University, 4-2 Aramaki, Maebashi, Gunma 371-8510, Japan; b Institute for Molecular and Celluar Regulation, Gunma University, 3-39-15 Shouwa, Maebashi 371-8512, Japan; c JASRI, 1-1-1 Kuoto, Sayo-cho, Sayo-gun, Hyogo 679-5198, Japan; d KEK-PF, 1-1 Oho, Tsukuba, Ibaraki 305-0801, Japan

**Keywords:** solution X-ray scattering, liposome, DDS

## Abstract

Lipid liposomes are promising drug delivery systems because they have superior curative effects owing to their high adaptability to a living body. Lipid liposomes encapsulating proteins were constructed and the structures examined using synchrotron radiation small- and wide-angle X-ray scattering (SR-SWAXS). The liposomes were prepared by a sequential combination of natural swelling, ultrasonic dispersion, freeze-throw, extrusion and spin-filtration. The liposomes were composed of acidic glycosphingolipid (ganglioside), cholesterol and phospholipids. By using shell-modeling methods, the asymmetric bilayer structure of the liposome and the encapsulation efficiency of proteins were determined. As well as other analytical techniques, SR-SWAXS and shell-modeling methods are shown to be a powerful tool for characterizing *in situ* structures of lipid liposomes as an important candidate of drug delivery systems.

## Abstract 2

Wide-angle X-ray scattering data using a third-generation synchrotron radiation source are presented.

## 1. Introduction

Liposomes are closed bilayer lipid systems and afford a very useful tool in various scientific fields including not only biology but also theoretical physics, biophysics, chemistry, colloid science and so on. In addition, during the past 30 years, liposomes have received a lot of attention as effective drug delivery systems (DDS) because they can reduce drug toxicity due to biodegradability and biocompatibility, and offer promising carriers of anti-cancer, anti-fungal and anti-biotic drugs, gene medicines and anesthetics and anti-inflammatory drugs, compared with other delivery systems (Kaneda, 2000 ▶; Rahimpour & Hamishehkar, 2012 ▶; Allen & Cullis, 2013 ▶). Liposome studies and those clinical trials have progressed from the use of conventional liposomes to that of ‘second-generation liposomes’ that are developed by modulating the lipid composition, size and modified surface of liposome (Torchilin, 2005 ▶). As a candidate reagent of surface modification of liposomes, glycolipids or sialic acids are considered to be promising.

On the other hand, the formation of a lipid microdomain in mammalian plasma membrane, a so-called lipid raft (Simons & Ikonen, 1997 ▶, 2000 ▶; Hakomori, 2001 ▶; Simons & Toomre, 2001 ▶), has been attracting intensive interest because lipid rafts are assumed to have functions as platforms of membrane-associated events such as signal transduction, cell adhesion, lipid/protein sorting and so on (Anderson & Jacobson, 2002 ▶; Gassart *et al.*, 2003 ▶; Helms & Zurzolo, 2004 ▶). A common feature of lipid rafts is their peculiar lipid composition, being rich in glycosphingolipids (GSLs), sphingomyelin and cholesterol. Gangliosides, major components of GSLs, are acidic lipids composed of a ceramide linked to an oligosaccharide chain containing one or more sialic acid residues, which are abundant in central nervous systems. Functions of lipid rafts are assumed to relate closely to the peculiar features of GSL molecules both in ceramide and oligosaccharide portions that can form complex hydrogen-bonding networks (hydrogen-bond donor and acceptor) (Pascher, 1976 ▶). Therefore, it can be assumed that gangliosides are promising reagents of liposome due to those intrinsic properties.

By using neutron and synchrotron X-ray scattering techniques [small-angle X-ray scattering (SAXS), small-angle neutron scattering (SANS)], we studied various functional properties of gangliosides and those aggregates with other lipids under various conditions. The temperature dependence of the hydration of the sugar head region and the dissociation degree of sialic acids (Hirai *et al.*, 1996*a* ▶,*b* ▶,*c* ▶, 1999 ▶; Hirai & Takizawa, 1998 ▶; Hayakawa & Hirai, 2002 ▶), the maximum miscibility of cholesterol molecules against gangliosides and the cholesterol-dependent micelle-to-vesicle transition (Hirai *et al.*, 2005 ▶), the vesicle-to-lamellar reversible transition by Ca^2+^ ions (Hayakawa & Hirai, 2003 ▶), and the asymmetric bilayer structures of ganglioside/cholesterol/phospholipid liposomes (Hirai *et al.*, 2003 ▶; 2006 ▶) were clarified. In addition, by using the neutron spin-echo technique, we found the function of ganglioside molecules controlling the undulation motion of micelles (Hirai *et al.*, 2001 ▶) and the bending modulus of liposomes (Hirai *et al.*, 2005 ▶). Recently, we have found that the interaction between ganglioside and amyloid beta protein (Aβ1–40) significantly reduces the diffusional bending motion of liposomes (Hirai *et al.*, 2013 ▶). These previous results show that lipid-rafts’ components (ganglioside/cholesterol-rich microdomains) can modulate properties of membrane structure and dynamics through the interaction between lipid and protein (hydrophobic coupling between a protein and a surrounding lipid bilayer) (Lundbaek *et al.*, 2004 ▶), suggesting that ganglioside molecules are useful for designing a new type of liposome DDS.

Based on the above results, we have studied the structural characteristics and stability of lipid-rafts model liposomes entrapping proteins by using synchrotron radiation small-angle and wide-angle X-ray scattering (SR-SWAXS). The liposomes investigated in the present study would be promising components of DDS because they are easily adjusted to attaining appropriate curative effects by modulating lipid composition, size and surface charge of liposomes due to their high and specific adaptability to a living body. In addition, in spite of numerous studies of liposomes, characterization of liposomes entrapping proteins, as far as we know, has been rarely carried out using scattering methods.

## 2. Materials and methods

### 2.1. Samples

The liposomes used were lipid mixtures composed of disialoganglioside (G_D1_) from bovine brains, cholesterol and phospholipids (egg-PC). Cholesterol was purchased from Sigma Chemical Co. (USA). G_D1_ and egg-PC were purchased from Avanti Polar Lipids Inc. (USA). The protein was myoglobin from horse skeletal muscle (Sigma). Liposome samples were prepared by the sequential combination of natural swelling, ultrasonic dispersion, freeze-throw, extrusion and spin-filtration, as described below. As a first step, G_D1_, phospholipid lipids and cholesterol were separately dissolved in a chloroform/methanol mixture solvent [1/1 (*v*/*v*)]. These solutions were mixed in the molar ratio [G_D1_]/[cholesterol]/[phospholipid] 0.1/0.1/1. After mixing, to remove the organic solvent, the lipid mixture solutions were dried under a nitrogen stream and annealed *in vacuo* overnight at 318 K. The dried mixtures were suspended in myoglobin solutions (1%, 2.5%, 5% *w*/*v* myoglobin in 10 m*M* Hepes buffer at pH 7.4), where the phospholipid concentration was 2% *w*/*v*. Those suspensions were stored all night to obtain giant multilamellar vesicles (GMLVs) by natural swelling. As a second step, the suspensions of GMLVs were sonicated for 10 min at ∼316 K to obtain homogeneous small unilamellar vesicles (SUVs) by using a high-power probe-type ultrasonicator (Model UH-50, SMT Co.). As a third step, these SUV solutions were subjected to a ten-time freezing–thawing process to obtain giant uni­lamellar vesicles (GUVs). As a fourth step, large unilamellar vesicle (LUV) solutions were prepared by an extrusion method using the LiposoFast Basic extruder system (Avestin, Canada) with a polycarbonate filter (pore diameter 100 nm). The samples were subjected to ∼50 passes through the filter. As a final step, the obtained LUVs solutions were subjected to spin-filtration for removing un-entrapped proteins and for concentrating LUVs by using an ultra-filtration device (Vivaspin-50K by Sartorius Co.) at 1000 r.p.m. for 10 min × 5 times. The validity of each step was checked by X-ray scattering measurement to optimize the above sample preparation condition.

### 2.2. X-ray scattering measurements

SR-SWAXS experiments were performed using the BL-40B2 spectrometer at the Japan Synchrotron Radiation Research Institute (JASRI, Harima, Japan) and by using the BL-10C spectrometer at the High Energy Accelerator Research Organization (KEK, Tsukuba, Japan). The X-ray wavelengths and the sample-to-detector distances were 0.75 Å for a 51 cm camera length and 1.0 Å for a 4089 cm camera length at BL-40B2, and 1.49 Å for a 190 cm camera length at BL-10C. X-ray scattering intensity was recorded using the R-AXIS IV IP detector from Rigaku. The exposure time was 10 s at BL-40B2 and 180 s at BL-10C. Each sample solution was contained in a cell composed of a pair of thin quartz windows with 1 mm path length and was served for SWAXS measurements.

### 2.3. Scattering data treatment and shell-modeling analysis

The background correction of wide-angle scattering data was described elsewhere (Hirai *et al.*, 2002 ▶, 2004 ▶). The distance distribution function, *p*(*r*), was obtained by Fourier transform of the observed scattering intensity, *I*(*q*), as
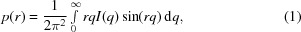
where *q* = (4π/λ)sin(θ/2), θ is the scattering angle and λ is the X-ray wavelength. We used the following model scattering function, *I*(*q*,*R*), of a multi-shelled ellipsoidal particle with a size distribution as shown previously (Hirai *et al.*, 2003 ▶),
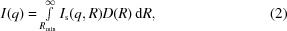
where *R*_min_ is the lower limit of the particle radius determined by the bilayer thickness of the LUV; *D*(*R*) is the number distribution function of the particle radius *R*; *I*_s_(*q*,*R*) is the spherical averaged scattering function of an ellipsoidal particle with radius *R* composed of *n* shells [*i*th shell with average excess scattering density 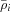 (so-called contrast), radius *R*_*i*_ and *V*_*i*_]. *I*_s_(*q*,*R*) and *D*(*R*) are given as follows,
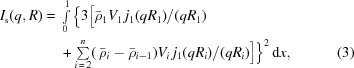
where *j*_1_ is the spherical Bessel function of the first rank. 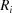 is defined by
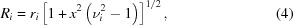
where *r*_*i*_ and *ν*_*i*_ are the semi-axis and its ratio of the *i*th ellipsoidal shell, respectively. For a spherical-shelled particle (*ν*_*i*_ = 1, *R*_*i*_ = *r*_*i*_), equation (3) is simplified to
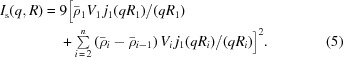
As a function of *D*(*R*), we adopted the Gaussian distribution function given by
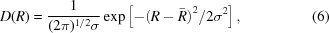
where 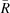 and σ are the average radius and the standard deviation, respectively.

## 3. Results and discussion

### 3.1. Removal of un-entrapped proteins from LUV solution by spin-filtration and its effect on LUV structure

**Figure 1:**
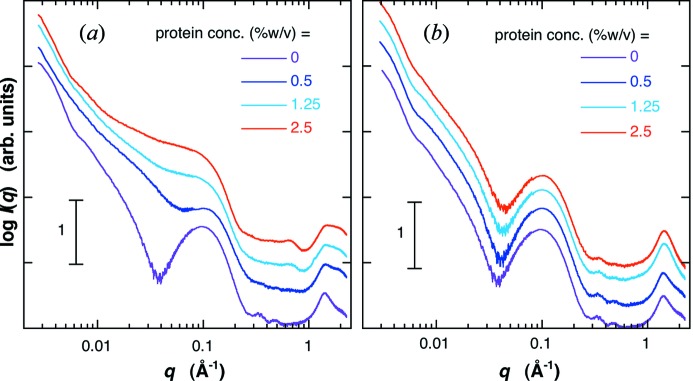
Scattering curves of the mixtures of LUV (un-entrapping proteins, [G_D1_]/[cholesterol]/[egg-PC] = 0.1/0.1/1) solution and protein solution at 298 K before and after spin-filtration for removing proteins existing in the external solution of LUV. (*a*) and (*b*) correspond to before and after spin-filtration, respectively. Before spin-filtration in (*a*), the protein concentrations were 0, 0.5, 1.25 and 2.5% (*w*/*v*), the LUV concentration 1.0% (*w*/*v*). The scattering curves are shifted along the vertical axis to avoid overlap.

**Figure 2:**
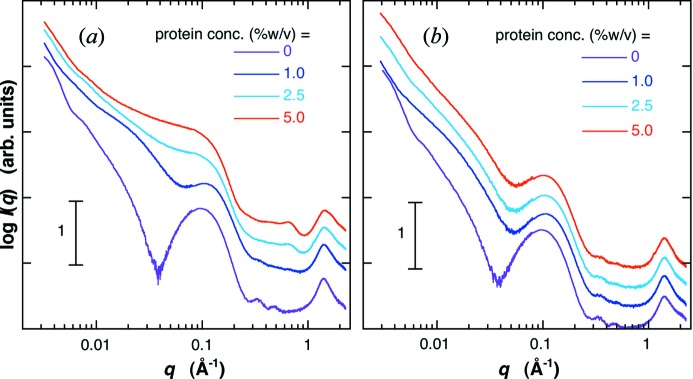
Scattering curves of LUVs entrapping proteins ([G_D1_]/[cholesterol]/[egg-PC] = 0.1/0.1/1) at 298 K before (*a*) and after (*b*) spin-filtration. The charged (initial) protein concentration was varied from 1% (*w*/*v*) to 5% (*w*/*v*), the LUV concentration 2.0% (*w*/*v*). The scattering curves are shifted along the vertical axis to avoid overlap.

In the present sample preparation, not only occlusion of proteins within LUVs but also removal of un-entrapped proteins from LUV solutions is important. Fig. 1 ▶ shows the scattering curves of the mixtures of LUV (un-entrapping proteins) solution and protein solution before (Fig. 1*a* ▶) and after (Fig. 1*b* ▶) spin-filtration. The molar ratio of lipids is [G_D1_]/[cholesterol]/[egg-PC] = 0.1/0.1/1. In Fig. 1(*a*) ▶, the concentration of the LUV was fixed to be 1.0% (*w*/*v*), and that of the protein was varied from 0.5 to 2.5% (*w*/*v*). As shown in Fig. 1(*a*) ▶, the presence of amounts of un-entrapped proteins in the external solution significantly changes the scattering curve in the *q* ranges of ∼0.01–0.15 Å^−1^ and of ∼1–2 Å^−1^. On the other hand, in spite of the addition of proteins with different concentrations, all scattering curves after spin-filtration agree with the scattering curve of LUV without the addition of proteins in the whole *q* range, indicating that un-entrapped (free) proteins were removed from the solution without affecting the LUV structure and its dispersity. Figs. 2(*a*) and 2(*b*) ▶ show the scattering curves of LUVs ([G_D1_]/[cholesterol]/[egg-PC] = 0.1/0.1/1) entrapped proteins before and after spin-filtration, respectively. The charged protein concentration was varied from 1% (*w*/*v*) to 5% (*w*/*v*). After spin-filtration, all scattering curves with different protein concentrations show a similar profile, suggesting that the proteins un-entrapped in LUVs were removed from the solutions. Hereinafter, we call LUV un-entrapped proteins and LUVs entrapped proteins empty-LUV and filled-LUV, respectively.

### 3.2. Structural characteristics of LUVs entrapping proteins

**Figure 3:**
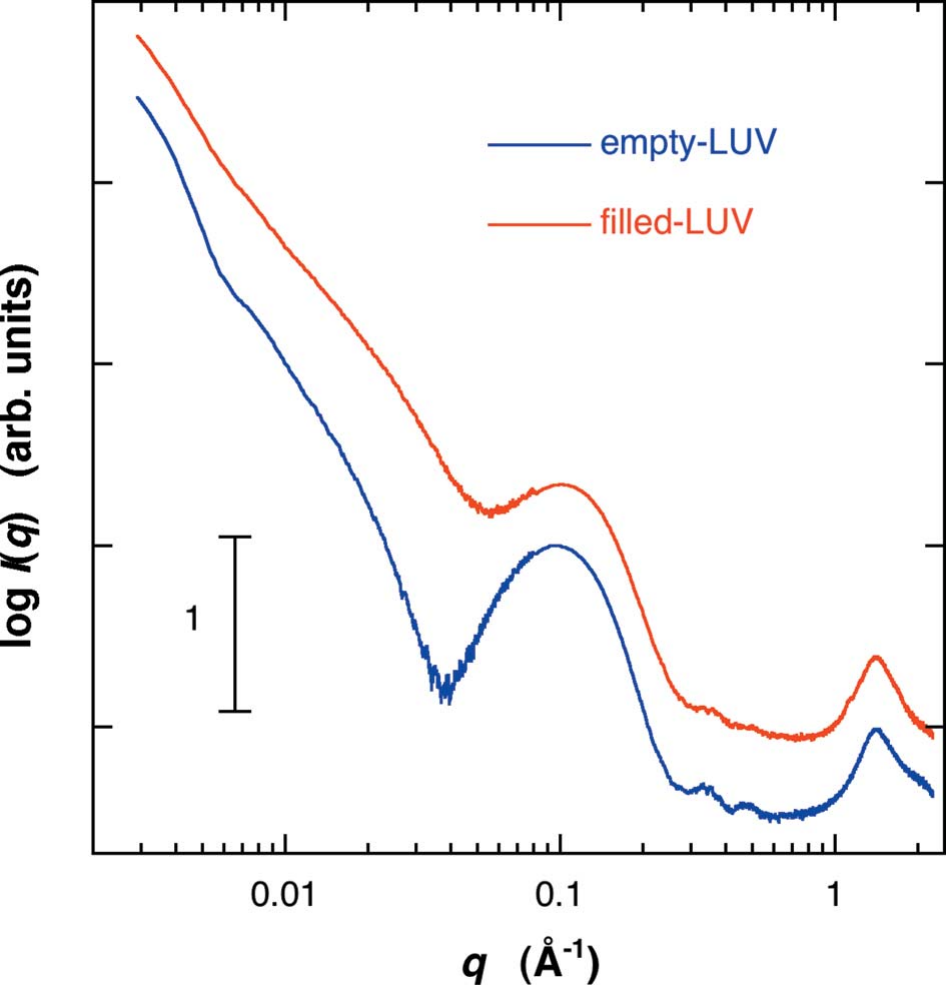
Comparison of the scattering curve of LUV un-entrapping proteins (empty-LUV) with that of entrapping ones (filled-LUV) at 298 K from Fig. 2 ▶ {[G_D1_]/[cholesterol]/[egg-PC] = 0.1/0.1/1, charged protein concentration was 5% (*w*/*v*)}.

**Figure 4:**
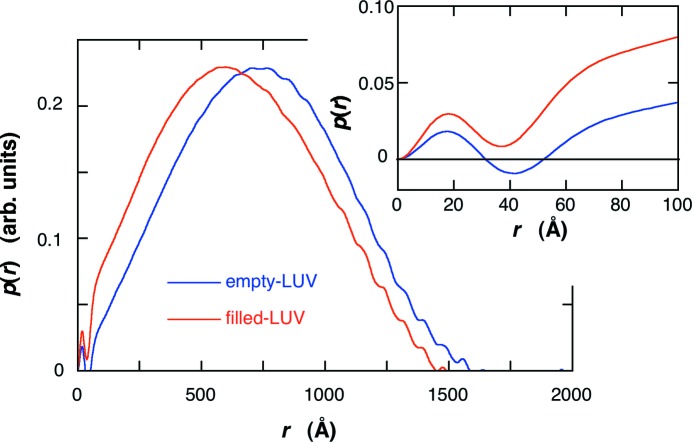
Comparison of the distance distribution function, *p*(*r*), of the empty-LUV with that of the filled-LUV obtained from Fig. 3 ▶. The insert expands the *p*(*r*) functions in the short-distance region.

Fig. 3 ▶ shows the scattering curves of the empty-LUV and the filled-LUV. The difference between the scattering curves appears in the *q* range of ∼0.01–0.15 Å^−1^, which is attributable to the change of the average excess scattering density (so-called contrast) of the core (water pool region) of the LUV by the protein occlusion, as shown in the following section. The effect of protein occlusion on the LUV structure is also seen in the distance distribution function, *p*(*r*), obtained by using equation (1). Fig. 4 ▶ shows the *p*(*r*) functions in the empty-LUV and the filled-LUV. The position of the maximum of the broad peak shifts from ∼715 Å for the empty-LUV to ∼590 Å for the filled-LUV, suggesting that the average contrast inside of the LUV becomes higher. In addition, there exists another evident difference in the *p*(*r*) functions at the short-distance region as shown in the insert in Fig. 4 ▶. The oscillation profile for the filled-LUV in Fig. 4 ▶ below ∼55 Å tends to be damped compared with that for the empty-LUV. The above changes in the scattering curves and in the *p*(*r*) functions are reasonably explained by the protein occlusion, as shown below.

### 3.3. Shell-modeling analysis and simulation of LUVs

**Figure 5:**
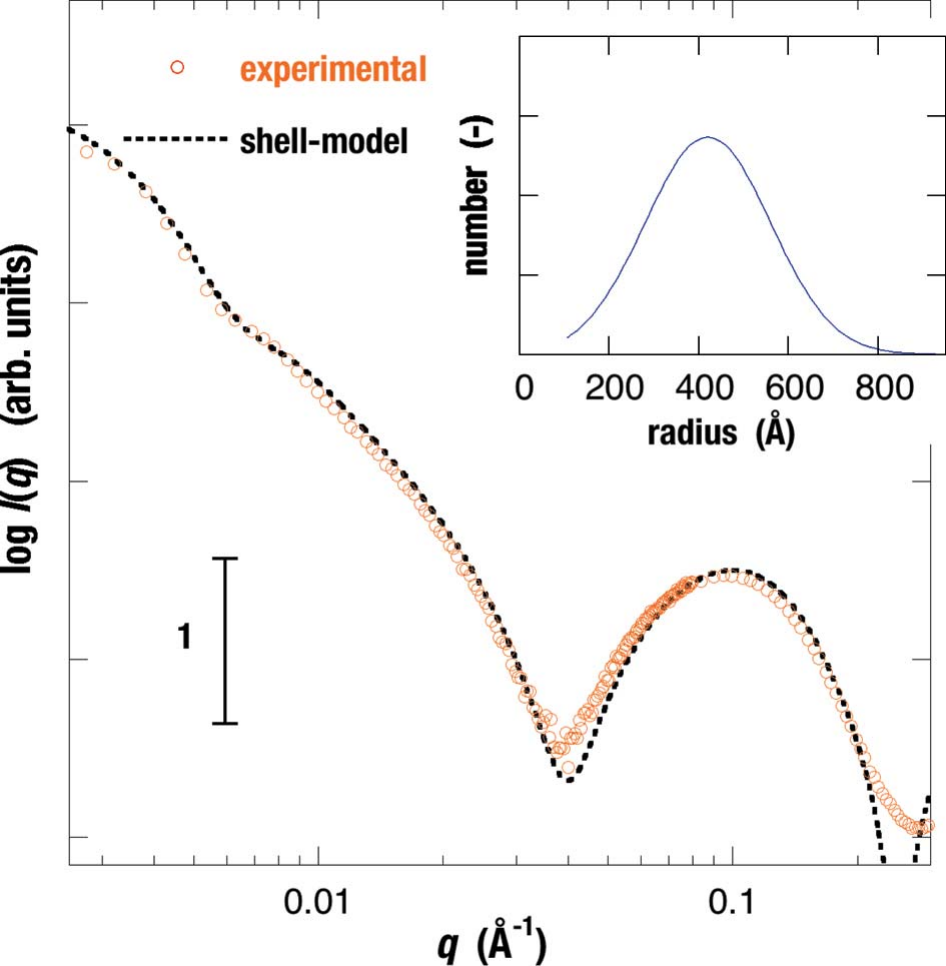
Experimental and theoretical scattering curves of the empty-LUV ([G_D1_]/[cholesterol]/[egg-PC] = 0.1/0.1/1) at 298 K using equations (2) and (5), where the insert is the obtained size distribution function. The reliability factor *R* is 0.051.

**Figure 6:**
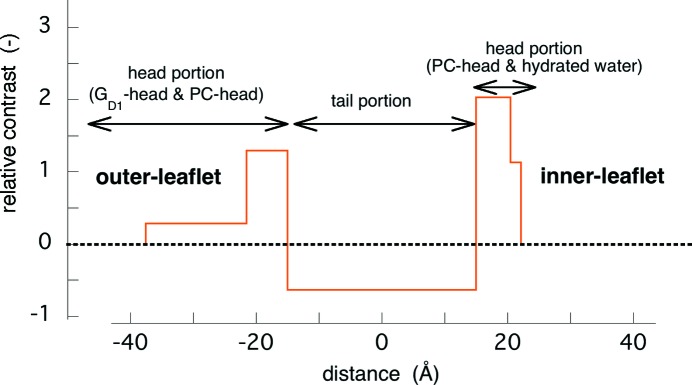
Structure of the bilayer of the empty-LUV obtained from the shell-modeling analysis in Fig. 5 ▶. The obtained value of contrast in each region is on a relative scale.

Owing to the simple structural symmetry of vesicles, the shell-model scattering function given by equations (2) and (5) is applicable for describing experimental scattering curves in many cases (Hirai *et al.*, 2003 ▶, 2013 ▶; Hirai, 2007 ▶; Onai & Hirai, 2010 ▶). Fig. 5 ▶ shows the experimental and theoretical scattering curves of the empty-LUV ([G_D1_]/[cholesterol]/[egg-PC] = 0.1/0.1/1), where the insert is the obtained size distribution function. The reliability factor *R* defined by *R* = Σ|*I*_experiment_(*q*) − *I*_theory_(*q*)|/Σ*I*_experiment_(*q*) is 0.051, which indicates the validity of the shell-model fitting. The deviation between the theoretical and experimental SAXS curves above *q* = ∼0.2 Å^−1^ is attributable to the simplification of the spherical shell structure. Fig. 6 ▶ shows the contrast profile of the intra-bilayer structure of the empty-LUV obtained from the modeling analysis. The low positive-contrast region at the outer-leaflet of the bilayer corresponds to the protruded portion of the oligosaccharide chains from the membrane surface, which is the common feature for LUVs containing ganglioside molecules (Hirai *et al.*, 2003 ▶, 2006 ▶, 2013 ▶; Hirai, 2007 ▶; Onai & Hirai, 2010 ▶). Such an asymmetry of the bilayer structure results from the difference in the geometrical packing parameters of lipid molecules. Namely, owing to the presence of the oligosaccharide chain containing sialic acid residues in the hydrophilic polar head region, gangliosides are preferentially localized at the outer-leaflet of the bilayer membrane to minimize the surface free energy. The high positive-contrast regions reflect the phosphocholine head portions at both sides. The positive-contrast region at the inner-leaflet of the bilayer corresponds to a high-density hydration shell around the phosphocholine heads.

**Figure 7:**
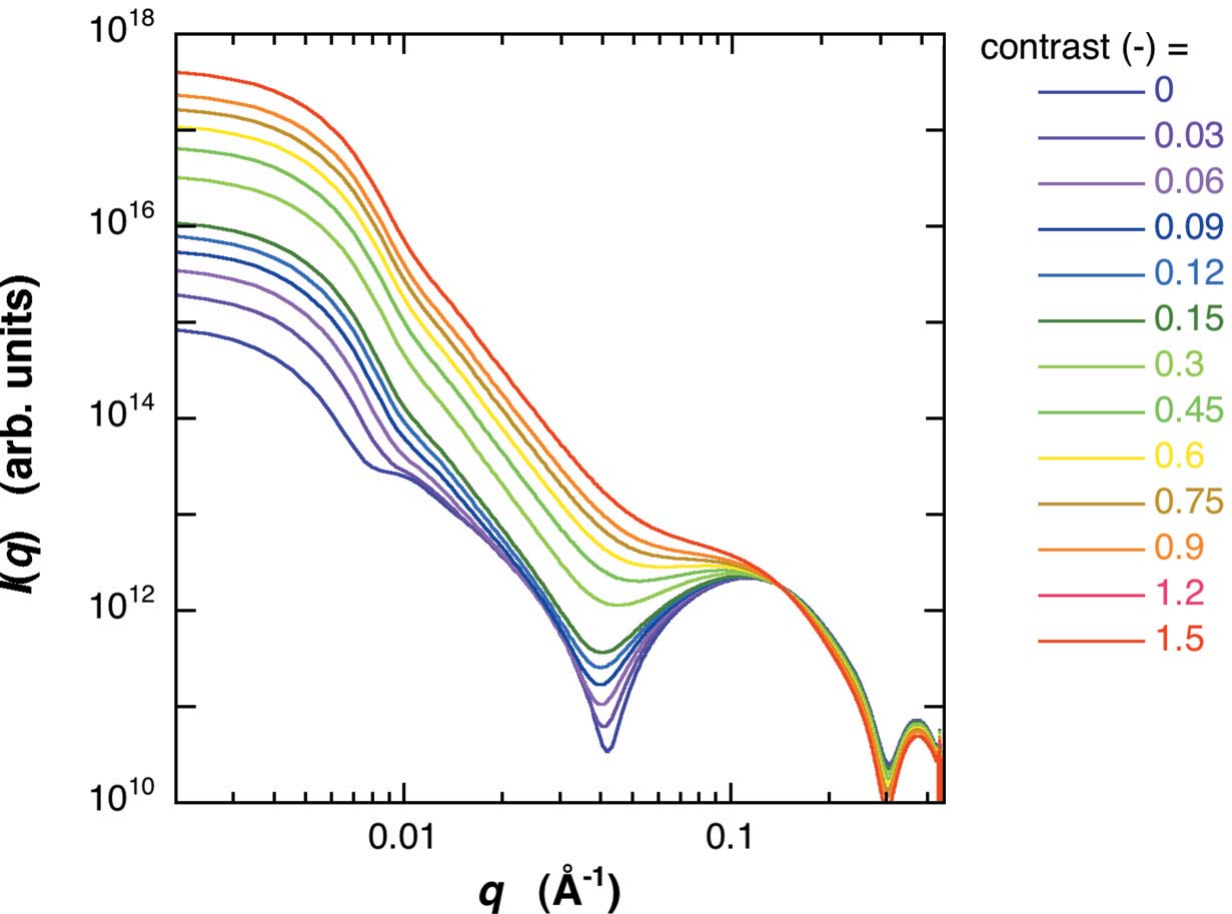
Shell-model simulation of LUV scattering function depending on the protein occlusion. The encapsulation efficiency of proteins [0 to ∼100% (*v*/*v*)] within the water pool of LUV corresponds to the change of the relative contrast of the innermost core of LUV from 0 to 2.6. The change of the scattering curve can explain the difference shown in Fig. 3 ▶.

**Figure 8:**
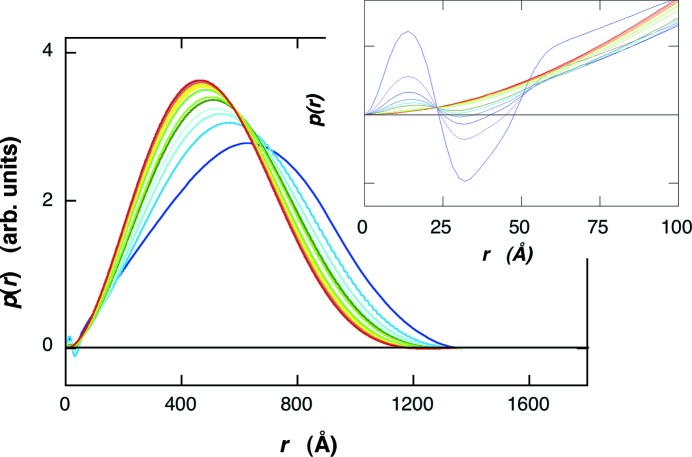
Simulated distance distribution function, *p*(*r*), of LUV depending on the protein occlusion, obtained from Fig. 7 ▶. The insert expands the *p*(*r*) functions in the short-distance region. The changes in the broad peak position at ∼500–700 Å and in the ripple profile below ∼55 Å can describe the experimental ones in Fig. 4 ▶.

Based on the above structural parameters of the open-LUV, we can simulate the effect of protein occlusion on the scattering curve of the LUV by using equations (1) and (5). The structure parameters of the bilayer were as in Fig. 6 ▶. The average radius and the standard deviation of the radius were 400 Å and 30%, respectively. The average scattering density of water for X-rays is 9.38 × 10^10^ cm^2^, and those of proteins range from ∼11.7 to ∼12.0 × 10^10^ cm^2^ (Stuhrmann & Miller, 1978 ▶). Therefore, the occlusion of proteins increases the contrast of the water pool of the LUV from zero to a positive value. Fig. 7 ▶ shows the shell-model scattering function of LUV with a size distribution depending on the change of the water-pool contrast accompanied by the protein occlusion. In Fig. 7 ▶ the relative value of the water-pool contrast was varied from 0 to 2.6, which corresponds to the encapsulation efficiency of the protein within the water-pool of LUV from 0 to ∼100% (*v*/*v*). As shown previously (Onai & Hirai, 2010 ▶), the scattering curve of an empty-LUV can be characterized by the shoulder at ∼0.01 Å^−1^ and the broad rounded peak at ∼0.04–0.2 Å^−1^. The former and the latter correspond to the spherical shape of LUVs with a size distribution (polydispersity) and to an internal structure of the bilayer, respectively. With ascending encapsulation efficiency, the scattering curve changes systematically, especially in the *q* range below ∼0.1 Å^−1^. The shoulder at *q* = ∼0.01 Å^−1^ and the dip at *q* = ∼0.04 Å^−1^ become smeared and/or shallower. These changes of the scattering curve can qualitatively explain the difference shown in Fig. 3 ▶. Fig. 8 ▶ shows the *p*(*r*) functions obtained from Fig. 7 ▶. With the rise of the water-pool contrast from 0 to 2.6 [the encapsulation efficiency of the protein from 0 to ∼100% (*v*/*v*)], the position of the peak maximum significantly shifts from ∼700 Å to ∼500 Å, and the ripple profile below ∼55 Å becomes smeared out as shown in the insert of Fig. 8 ▶. The above results of the shell-modeling simulation qualitatively describe the tendency of the observed changes in Figs. 3 ▶ and 4 ▶.

### 3.4. Estimation of encapsulation efficiency of proteins

**Figure 9:**
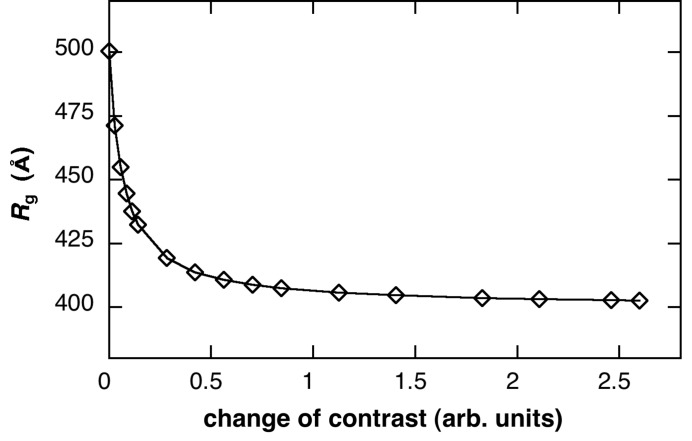
Radius of gyration, *R*_g_, of LUV depending on the protein occlusion, which was obtained from the simulated scattering functions in Fig. 8 ▶. The *R*_g_ value is plotted against the change of the relative contrast of the innermost core of the LUV.

**Figure 10:**
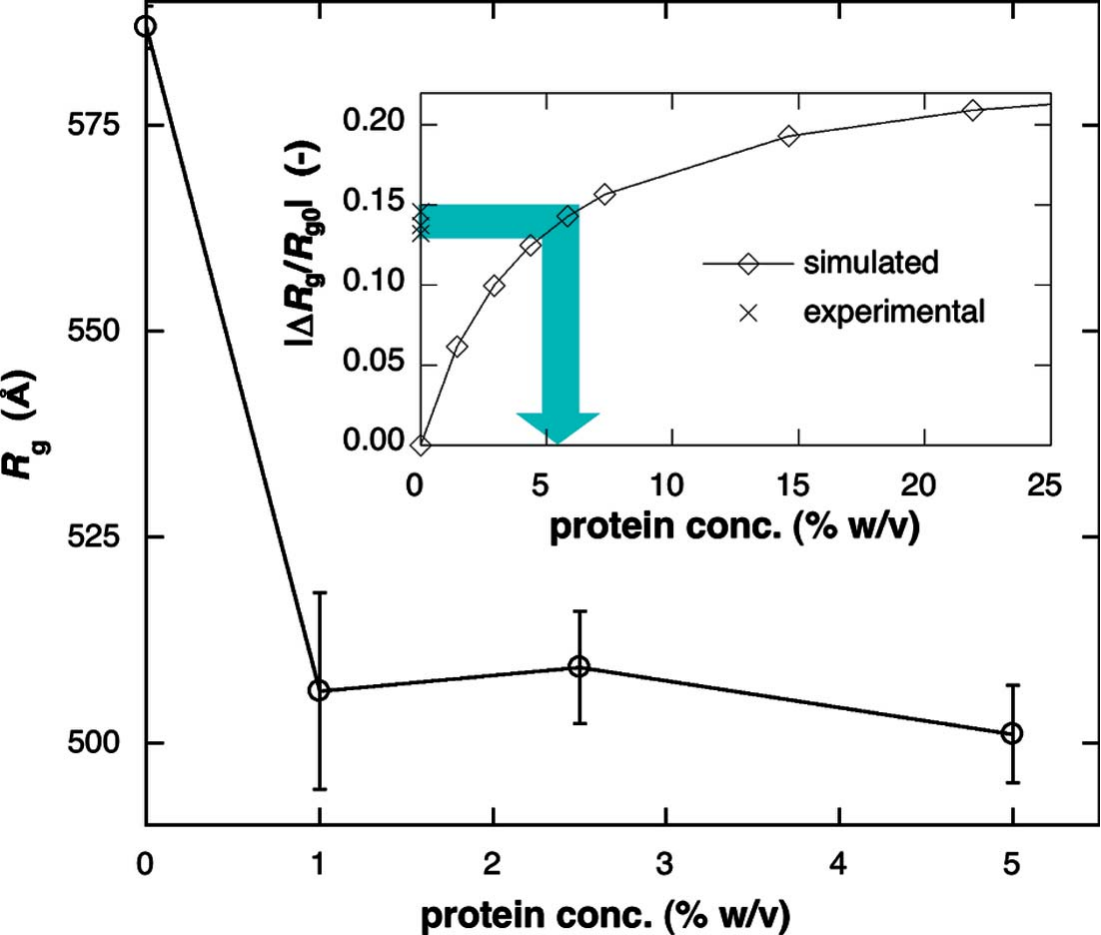
Experimental radii of gyration, *R*_g_, of filled-LUVs for the different charged amounts of proteins within the LUVs from 1% (*w*/*v*) to 5% (*w*/*v*). The insert shows the relation between the encapsulated protein concentration [% (*w*/*v*)] and the decrement rate of *R*_g_ obtained from Fig. 8 ▶, which was used to estimate the encapsulated protein concentration.

Radii of gyration obtained from small-angle X-ray scattering data using Guinier plots (Guinier & Fournet, 1955 ▶) are well known to be useful for characterizing structures of particles with random orientation. Fig. 9 ▶ shows the water-pool contrast dependence of the radius of gyration, *R*_g_, of LUV obtained from Fig. 7 ▶. In Fig. 9 ▶, the *R*_g_ of LUV decreases sensitively against the rise of the contrast (encapsulation efficiency), especially at low protein occupancy. In the present sample preparation, we varied the charged amount of proteins within the LUV from 1% (*w*/*v*) to 5% (*w*/*v*). Fig. 10 ▶ shows the experimental *R*_g_ values of the LUVs obtained from Fig. 2(*b*) ▶ for the different charged amount of proteins. The *R*_g_ value decreased from 587 ± 3 Å for the empty-LUV to 506 ± 9 Å [1% (*w*/*v*)], to 509 ± 7 Å [2.5% (*w*/*v*)] and to 501 ± 6 Å [5.0% (*w*/*v*)] for the filled-LUVs. The experimental *R*_g_ values of the filled-LUVs are similar within experimental error. The insert of Fig. 10 ▶ shows the simulated relation between the encapsulated protein concentration [% (*w*/*v*)] and the decrement rate of *R*_g_ of filled-LUV normalized by *R*_g_ of the empty-LUV. From this relation, we can determine the encapsulated protein concentrations of the filled-LUVs to be ∼5.5% (*w*/*v*). In spite of the difference of the initial protein concentration, the encapsulation efficiency tends to be higher than the charged amount of proteins, suggesting that the present LUV preparation induces a slight condensation of proteins within the water-pool of the LUV.

## 4. Conclusion

By the combination of different types of liposome preparation methods, proteins were effectively encapsulated within the water pool of LUV composed of glycosphingolipid (ganglioside G_D1_), cholesterol and phospholipid. Ganglioside molecules would be promising reagents for designing a new type of liposome DDS since gangliosides can modulate the size and surface charge of liposomes owing to these intrinsic properties. The observed changes in the SWAXS curve of the LUV caused by the encapsulation of proteins were reasonably explained by the shell-model simulation. The encapsulation efficiency of proteins within LUV was also determined, suggesting that the concentration of the encapsulated proteins tends to be higher than that of the initially charged proteins (condensation effect). This would result from the LUV preparation method used here. On the other hand, recently, different analytical techniques, such as scanning probe microscope, fluorescent probe microscopy and so on (Spyratou *et al.*, 2009 ▶), can be applied to characterize liposomes. As well as other techniques, the present results show that structures of LUVs of lipid mixtures as an important candidate of DDSs can be characterized well by using SR-SWAXS and shell-modeling methods.

## References

[bb1] Allen, T. M. & Cullis, P. R. (2013). *Adv. Drug Deliv. Rev.* **65**, 36–48. 10.1016/j.addr.2012.09.037 23036225

[bb2] Anderson, R. G. & Jacobson, K. (2002). *Science*, **296**, 1821–1825. 10.1126/science.1068886 12052946

[bb3] Gassart, A. de, Geminard, C., Fevrier, B., Raposo, G. & Vidal, M. (2003). *Blood*, **102**, 4336–4344. 10.1182/blood-2003-03-0871 12881314

[bb4] Guinier, A. & Fournet, G. (1955). *Small-Angle Scattering of X-rays.* New York: John Wiley.

[bb5] Hakomori, S. (2001). *Trends Glycosci. Glycotechnol.* **13**, 219–230.

[bb6] Hayakawa, T. & Hirai, M. (2002). *Eur. Biophys. J.* **31**, 62–72. 10.1007/s00249-001-0190-2 12046898

[bb7] Hayakawa, T. & Hirai, M. (2003). *J. Appl. Cryst.* **36**, 489–493.

[bb8] Helms, J. B. & Zurzolo, C. (2004). *Traffic*, **5**, 247–254. 10.1111/j.1600-0854.2004.0181.x 15030566

[bb33] Hirai, M. (2007). *J. Phys. Conf. Ser.* **83**, 012003.

[bb9] Hirai, M., Hirai, H., Koizumi, M., Kasahara, K., Yuyama, K. & Suzuki, N. (2006). *Physica B*, **385**–**38**6, 868–870.

[bb10] Hirai, M., Iwase, H. & Hayakawa, T. (1999). *J. Phys. Chem. B*, **103**, 10136–10142.

[bb11] Hirai, M., Iwase, H. & Hayakawa, T. (2001). *J. Phys. Soc. Jpn*, **70**, s420–s423.

[bb12] Hirai, M., Iwase, H., Hayakawa, T., Koizumi, M. & Takahashi, H. (2003). *Biophys. J.* **85**, 1600–1610. 10.1016/S0006-3495(03)74591-3 PMC1303335 12944276

[bb13] Hirai, M., Iwase, H., Hayakawa, T., Miura, K. & Inoue, K. (2002). *J. Synchrotron Rad.* **9**, 202–205. 10.1107/s0909049502006593 12091726

[bb14] Hirai, M., Kimura, R., Takeuchi, K., Sugiyama, M., Kasahara, K., Ohta, N., Farago, B., Stadler, A. & Zaccai, G. (2013). *Eur. Phys. J. E*, **36**, 74. 10.1140/epje/i2013-13074-3 23852578

[bb15] Hirai, M., Koizumi, M., Hayakawa, T., Takahashi, H., Abe, S., Hirai, H., Miura, K. & Inoue, K. (2004). *Biochemistry*, **43**, 9036–9049. 10.1021/bi0499664 15248761

[bb16] Hirai, M., Koizumi, M., Hirai, H., Hayakawa, T., Yuyama, K., Suzuki, N. & Kasahara, K. (2005). *J. Phys. Condens. Matter*, **17**, S2965–S2977.

[bb17] Hirai, M. & Takizawa, T. (1998). *Biophys. J.* **74**, 3010–3014. 10.1016/S0006-3495(98)78008-7 PMC1299642 9635755

[bb18] Hirai, M., Takizawa, T., Yabuki, Y. & Hayashi, K. (1996*b*). *J. Chem. Soc. Faraday Trans.* **92**, 4533–4540.

[bb19] Hirai, M., Takizawa, T., Yabuki, S., Hirai, T. & Hayashi, K. (1996*c*). *J. Phys. Chem.* **100**, 11675–11680.

[bb20] Hirai, M., Takizawa, T., Yabuki, S., Nakata, Y. & Hayashi, K. (1996*a*). *Biophys. J.* **70**, 1761–1768. 10.1016/S0006-3495(96)79739-4 PMC1225145 8785335

[bb21] Kaneda, Y. (2000). *Adv. Drug Deliv. Rev.* **43**, 197–205. 10.1016/s0169-409x(00)00069-7 10967226

[bb22] Lundbaek, J. A., Birn, P., Hansen, A. J., Søgaard, R., Nielsen, C., Girshman, J., Bruno, M. J., Tape, S. E., Egebjerg, J., Greathouse, D. V., Mattice, G. L., Koeppe, R. E. & Andersen, O. S. (2004). *J. Gen. Physiol.* **123**, 599–621. 10.1085/jgp.200308996 PMC2234500 15111647

[bb24] Onai, T. & Hirai, M. (2010). *J. Phys. Conf. Ser.* **247**, 012018.

[bb25] Pascher, I. (1976). *Biochem. Biophys. Acta*, **455**, 433–451. 10.1016/0005-2736(76)90316-3 999922

[bb26] Rahimpour, Y. & Hamishehkar, H. (2012). *Expert Opin. Drug Deliv.* **9**, 443–455. 10.1517/17425247.2012.666968 22413847

[bb27] Simons, K. & Ikonen, E. (1997). *Nature (London)*, **387**, 569–572. 10.1038/42408 9177342

[bb28] Simons, K. & Ikonen, E. (2000). *Science*, **290**, 1721–1726. 10.1126/science.290.5497.1721 11099405

[bb29] Simons, K. & Toomre, D. (2001). *Nat. Rev. Mol. Cell Biol.* **2**, 216. 10.1038/35036052 11413487

[bb30] Spyratou, E., Mourelatou, E. A., Makropoulou, M. & Demetzos, C. (2009). *Expert Opin. Drug Deliv.* **6**, 305–317. 10.1517/17425240902828312 19327046

[bb31] Stuhrmann, H. B. & Miller, A. (1978). *J. Appl. Cryst.* **11**, 325–345.

[bb32] Torchilin, V. P. (2005). *Nat. Rev. Drug Discov.* **4**, 145–160. 10.1038/nrd1632 15688077

